# Feasibility and perceived educational value of extended reality trauma simulation for emergency medical teams in a multinational training context

**DOI:** 10.1186/s41077-026-00418-x

**Published:** 2026-08-18

**Authors:** Linda Sonesson, Kenneth D Boffard, Gabriel Reedy

**Affiliations:** 1 Florence Nightingale Faculty of Nursing, Midwifery and Palliative Care, King’s College London, London, UK; 2 Milpark Hospital Academic Trauma Center, Department of Surgery, University of the Witwatersrand, 17 Pallinghurst Road Parktown, Johannesburg, ZA 2193 South Africa; 3 Faculty of Life Sciences and Medicine, King’s College London, Waterloo Bridge Wing G7, London, UK

**Keywords:** Extended reality, Educational feasibility, Simulation-based education, Emergency medical teams, Trauma education

## Abstract

**Background:**

Large-scale civil military medical live simulation exercises such as NATO Centre of Excellence for Military Medicine’s (NATO MILMED COE) Vigorous Warrior (VW) series provide authentic environments to test interoperability and clinical performance of Emergency Medical Teams (EMTs). However, such exercises are resource intensive, logistically demanding, and infrequent, raising challenges for sustained workforce readiness. Extended Reality (XR) has emerged as a complementary modality with potential to extend access to trauma training, reduce costs, and support distributed learning. This study aimed to examine the educational feasibility of XR trauma simulation when embedded in multinational large scale live simulation.

**Methods:**

We conducted an educational feasibility study during Vigorous Warrior 2024 in Hungary, embedded within NATO MILMED COE’s multinational large scale medical live simulation. Twenty-five EMT members from three countries (physicians, anaesthesiologists, nurses, and radiographers) participated in an XR-based penetrating chest trauma scenario using the MilExTS XR platform (Meta Quest 3 headset). Feasibility outcomes were evaluated across recruitment, implementation, usability, acceptability and perceived educational value using session logs, post-simulation surveys (Likert scales 1–5), and open-ended qualitative feedback. Data were analysed descriptively and thematically, in line with published feasibility frameworks for medical education.

**Results:**

Recruitment and completion thresholds were fully achieved (100% participation, 100% survey completion). Acceptability was high, with 88% of participants rating XR as good or excellent for trauma training and indicating willingness to use it in future education. Two-thirds (66%) endorsed XR for enhancing teamwork, while 68% valued opportunities for individual decision-making practice in a safe environment. Usability findings were mixed: 56% of participants rated scenario realism as average or below, cited limited physiological fidelity, lack of tactile feedback, headset navigation challenges, and insufficient role differentiation. Qualitative data supported XR’s perceived educational value while identifying areas requiring further technological and pedagogical development.

**Conclusion:**

XR trauma simulation demonstrated educational feasibility, high acceptability, and perceived learning value when embedded within a complex multinational large scale live simulation exercise. While limitations in realism and interface design remain, XR shows promise as a complementary modality for interprofessional trauma training. Further research should progress to efficacy testing, cost-effective analysis and curricular integration before wider adoption.

**Supplementary Information:**

The online version contains supplementary material available at https://doi.org/10.1186/s41077-026-00418-x.

## Background

Emergency Medical Teams (EMTs) are mandated to deliver safe, coordinated, and high-quality care under surge conditions, often in multinational deployments and austere environments. Building and sustaining this capability has been identified as a core priority in the *World Health Organization Emergency Medical Teams (EMT) Strategy 2030*, which emphasises education, simulation-based training, and readiness assessment as essential components of workforce development and system preparedness [1]. Within NATO, large-scale live civil-military medical simulation exercises such as the *Vigorous Warrior* (VW) series provide authentic training environments to evaluate interoperability, coordination, and clinical performance across civil-military teams [2, 3]. While such exercises represent the pinnacle of experiential fidelity, they require significant logistical and financial investment and occur infrequently, limiting opportunities for continuous professional development, particularly for dual-role clinicians working across civilian and military systems [2–4]. Despite growing interest in immersive technologies for trauma training, few feasibility studies have examined the implementation of Extended Reality (XR) embedded within large-scale, live multinational simulation exercises, as opposed to controlled simulation-centre environments.

Simulation-based education has repeatedly demonstrated its effectiveness in improving both technical and non-technical skills in emergency and trauma care. Systematic reviews reaffirm its role in preparing clinicians for high-acuity, low-frequency events and in enhancing crisis resource management competencies [4–8]. However, traditional live simulations are constrained by scalability, accessibility, and sustainability, highlighting the need for complementary approaches that extend the reach and efficiency of training without compromising educational integrity.

Emerging immersive technologies, including Virtual Reality (VR), Augmented Reality (AR), and Extended Reality (XR), are increasingly used to complement traditional simulation in trauma and emergency medicine education. Prior studies report improved learner engagement, confidence, and perceived preparedness, particularly for high-acuity, low-frequency scenarios [9–13]. However, existing research has largely been conducted in controlled academic or simulation-centre settings, with limited attention to usability, acceptability, and implementation challenges in operational environments. Reported limitations include variable fidelity, usability barriers, and challenges integrating XR into established training systems, highlighting the need for feasibility-focused research conducted under authentic training conditions.

Empirical findings from NATO and allied defence medical education programmes have begun to demonstrate the educational potential of virtual and extended reality trauma simulations for enhancing Emergency Medical Team (EMT) readiness and interprofessional coordination [2, 3, 13]. Controlled evaluations within these initiatives have reported improvements in participants’ perceived preparedness, situational awareness, and teamwork effectiveness during complex, large-scale live simulation exercises [4, 8]. However, the degree of educational benefit appears to vary according to participants’ clinical experience, technological familiarity, and contextual factors such as operational tempo and team composition [2, 4]. These studies collectively highlight both the promise and current limitations of Extended Reality (XR) applications in trauma education: while immersive technologies can strengthen confidence, collaboration, and perceived readiness, they continue to face challenges in achieving high physiological fidelity, realistic role differentiation, and full alignment with evidence-based clinical and tactical protocols. This study therefore applied an educational feasibility framework to evaluate the implementation, usability, acceptability, and perceived educational value of XR trauma simulation during NATO MILMED COE’s Vigorous Warrior 2024 multinational live exercise. By examining the educational feasibility of XR within a multinational live simulation exercise, this study provides evidence of relevance to simulation educators, curriculum designers, and programme leaders seeking scalable, context-sensitive approaches to interprofessional trauma training.

## Methods

### Study design

This study was designed as an educational feasibility study, focusing on the practicality, acceptability, and perceived educational value of Extended Reality (XR) trauma simulation rather than on learning effectiveness or clinical outcomes. Feasibility was conceptualised using established frameworks for early-stage educational interventions, which emphasise recruitment, implementation, usability, acceptability, and perceived value as prerequisites for subsequent efficacy testing [11–13]. This approach is particularly appropriate for immersive educational technologies deployed in complex, real-world training environments, where contextual constraints and user experience strongly influence viability and scalability. We adopted a mixed-method educational feasibility design integrating quantitative and qualitative data to evaluate the implementation, acceptability, usability and perceived educational value of a novel XR simulation embedded within the NATO MILMED COE VW24 multinational live simulation exercise. Feasibility studies are recommended when an intervention is novel, delivered in operational complex settings, or requires assessment of implementation parameters prior to formal outcome evaluation [11–13]. The study design was guided by published frameworks for feasibility research in medical education [14], which emphasise the integration of quantitative indicators (e.g. recruitment, completion, usability ratings) with qualitative exploration of participant experience. Quantitative data were used to describe feasibility outcomes, while qualitative data from participant free-text responses and facilitator observations were analysed to identify perceived strengths, limitations, and contextual influences. This triangulated approach enabled a comprehensive assessment of XR feasibility within an authentic multinational trauma training context.

### Participants

Emergency Medical Teams (EMTs) participating in the NATO MILMED COE VW24 large scale live simulation exercise was prospectively identified by national commanders and invited to take part in the study. Eligibility criteria included active participation in the exercise, current clinical practice in emergency medicine, trauma, or critical care, and sufficient English proficiency to engage with the XR simulation interface and study materials. No prior experience with Extended Reality (XR) technology was required.

Recruitment procedures reflected the operational context of a multinational military exercise and were designed to support ecological validity by including a multidisciplinary mix of professional roles representative of deployed EMTs. Participant numbers and demographic characteristics are reported in the Results section.

### Study context

The study was embedded within Vigorous Warrior 2024 (VW24), NATO MILMED COE’s largest civil-military medical interoperability exercise, conducted in Hungary. VW24 involved approximately 1,900 participants from 36 countries and was designed to enhance preparedness and interoperability during large-scale emergency preparedness and disaster response.

The Concept Development and Experimentation (CD&E) track incorporated in the VW24, provides a structured platform for research and innovation conducted under operationally realistic conditions. This study formed part of the CD&E programme and was delivered in parallel with live simulation activities. Embedding the research within this context enabled evaluation of XR trauma simulation under authentic logistical, temporal, and organisational constraints typical of multinational emergency medical deployments.

### The XR technology and simulation platform

**Fig. 1:**
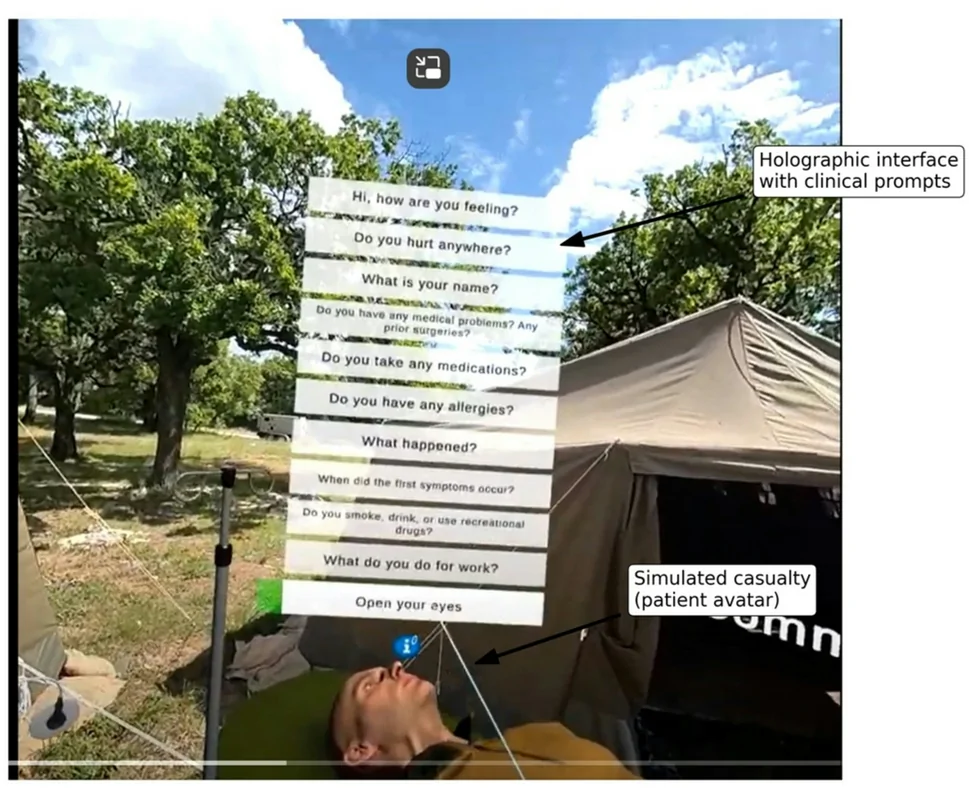
Annotated screenshot of the XR trauma simulation environment

The XR trauma simulation was delivered using MilExTS (Exonicus Inc.), a Tactical Combat Casualty Care (TCCC)-aligned training platform designed to support team-based trauma education. The simulation immersed participants in a Role 1 military field medical environment, defined as a forward emergency care facility providing immediate, life-saving interventions prior to evacuation (Fig. 1). This setting was selected to reflect the clinical and logistical constraints commonly encountered by Emergency Medical Teams operating in austere or deployed contexts.

The scenario focused on the management of penetrating chest trauma and incorporated dynamic physiological responses to clinical interventions. Participants had access to virtual clinical resources, including patient notes, monitoring data, radiology images, laboratory results, and procedural guidance. Interactive holographic visualisations and clinical prompts supported assessment, history-taking, shared situational awareness, and team-based clinical reasoning.

The simulation was accessed using the Meta Quest 3 mixed-reality headset, an untethered device selected for its portability and suitability for deployment in field conditions. Multiplayer functionality enabled synchronous participation and communication between team members within the XR environment. The platform was intended to support experiential learning, team coordination, and decision-making under time pressure rather than to replicate tactile procedural performance.

### Procedures

Participants engaged in the XR trauma simulation through a structured sequence comprising orientation, scenario enactment, facilitated oversight, and post-session debriefing with survey evaluation.

### Orientation

Participants first attended a brief, facilitated orientation designed to familiarise them with the XR hardware and software interface. This included headset calibration, navigation of clinical data, and practice using interactive three-dimensional visualisation tools. The orientation was intentionally concise to reflect the operational constraints of field deployment while ensuring that first-time users could engage effectively with the system.

### Scenario

Teams then participated in a trauma resuscitation scenario involving a patient with a penetrating chest trauma injury. Each XR scenario was undertaken by small interprofessional teams comprising 4–6 participants. Teams were formed pragmatically within the operational constraints of the exercise and were designed to reflect deployed Emergency Medical Team practice. The teams included a mix of professionals (physicians, anaesthesiologists, nurses, and radiographers) and nationalities, enabling exploration of interprofessional and multinational collaboration within the XR environment.

Scenario design was informed by learning objectives from Advanced Life Support (ALS) and Tactical Combat Casualty Care (TCCC) training frameworks. Participants were required to assess the patient, initiate resuscitation, and manage the evolving clinical condition, including invasive interventions such as chest decompression, airway management, and fluid resuscitation when clinically indicated.

The scenario was designed to examine both individual clinical reasoning and interprofessional team coordination under time pressure within the XR environment.

### Facilitation

Sessions were overseen by experienced simulation practitioners and members of the research team. Facilitators provided technical support to ensure the safe and effective operation of the XR platform but deliberately refrained from intervening in clinical decision-making. This approach was intended to preserve the authenticity of the scenario and enable observation of unprompted team interactions within the XR environment.

### Debriefing and survey

At the conclusion of each session, participants engaged in a brief, facilitated debriefing focusing on team communication, role clarity, and clinical decision-making. The debriefing was supported by the Plus- DELTA simulation framework [15] and structured to support reflective discussion and collective sense-making and was adapted to the operational constraints of a live exercise environment, while remaining consistent with core principles outlined in the INACSL Standards of Best Practice for Debriefing [16].

Immediately following the debriefing, participants completed an individual post-session survey designed to capture feasibility outcomes related to usability, acceptability, and perceived educational value. The survey incorporated both Likert-scale items and open-text fields to explore participant perceptions of realism, learning relevance, and acceptance of XR as a training modality.

### Ethical considerations

Ethical approval for the study was obtained from the NATO MILMED COE, which served as the primary sponsor, and from the Research Ethics Office at King’s College London (Ref: LRS-23/24-42892). The dual approval process reflected the cross-institutional and international character of the research, ensuring compliance with both military governance structures and academic standards for human subjects’ research.

All participants received written information describing the purpose, procedures, and voluntary nature of the study prior to enrolment. Informed consent was obtained through national team commanders, in accordance with NATO research governance protocols for multinational military exercises. This approach ensured that recruitment and participation were consistent with operational command structures while upholding ethical principles of autonomy and transparency.

Survey responses were anonymised, and no identifiable clinical or operational data were collected. Participation in the XR study was voluntary and did not influence participants’ roles, responsibilities, or evaluation within the wider VW24 exercise. These measures were implemented to minimise the risk of coercion and to protect participant confidentiality and well-being.

### Data collection

Data were collected from multiple complementary sources to evaluate feasibility outcomes across implementation, usability, acceptability, and perceived educational value.

### Observational data

Structured session logs (Appendix 1) were maintained by the research team to capture the duration of each XR session, the occurrence and resolution of technical issues, and overall participant engagement. These logs served as objective indicators of feasibility and implementation fidelity.

### Survey data

Following completion of the simulation and debrief, each participant was asked to complete an individual post-session survey (Appendix 2). The survey contained both quantitative and qualitative elements:

Quantitative items consisted of structured questions rated on a five-point Likert scale (1 = strongly disagree, 2 = disagree, 3 = neutral, 4 = agree, 5 = strongly agree). These items assessed domains including ease of use of the XR device and software, perceived learning value, confidence in clinical decision-making, realism of the simulation, and willingness to use XR in future training.

Qualitative items included open-text fields that invited participants to elaborate on their experiences, identify perceived strengths and weaknesses of the XR platform, and suggest potential improvements. These narrative responses provided rich contextual insights to complement quantitative ratings.

Survey items were purpose-designed for this study, informed by prior literature on XR usability and simulation-based education, and reviewed by the research team for face validity. Formal psychometric validation was beyond the scope of this feasibility study.

### Contextual data

Additional demographic and experiential variables were recorded to aid interpretation of survey responses. These included team composition, participants’ clinical role, and prior experience with simulation-based education. Such contextual data allowed exploration of how professional background and simulation familiarity might influence participants’ perceptions of XR usability and educational value.

### Data analysis

A mixed-methods analytic approach was adopted to address the multidimensional nature of feasibility outcomes.

### Quantitative analysis

Quantitative survey data were analysed descriptively to examine feasibility outcomes across predefined domains, including recruitment, completion, usability, acceptability, and perceived educational value. Predefined feasibility thresholds, aligned with established guidance for pilot and feasibility research, were applied to interpret outcomes and to determine whether criteria for feasibility were achieved. [11–13]. Descriptive analyses were performed without inferential statistical testing, consistent with the exploratory aims of feasibility studies.

### Qualitative analysis

Narrative data from free-text survey responses and facilitator field notes were analysed thematically. An inductive coding strategy was applied, enabling categories and themes to emerge directly from participant accounts rather than from a priori assumptions. Coding was undertaken independently by two members of the research team to enhance credibility and reduce bias. Emergent themes were compared, and discrepancies were resolved through iterative discussion and consensus-building. This process provided a nuanced understanding of participants’ perspectives on usability, educational value, and barriers or facilitators to XR adoption.

### Integration of findings

Triangulation of quantitative and qualitative results was undertaken to enhance validity and provide a comprehensive view of feasibility outcomes. The integration of descriptive metrics with experiential themes allowed convergence and complementarity of findings, ensuring that both measurable outcomes and contextual insights informed interpretation.

### Analytical rigour

**Fig. 2:**
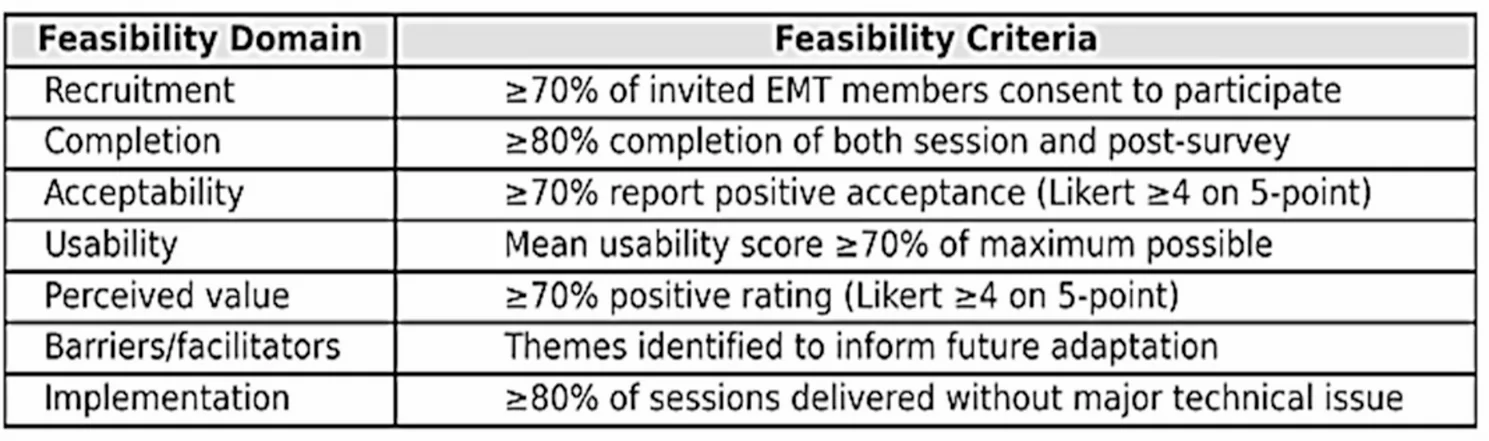
Feasibility outcomes framework guiding data collection and analysis

All analyses were conducted independently by members of the research team, with cross-checking procedures implemented to ensure accuracy and reliability of the findings. The evaluation was structured around the feasibility outcomes framework, presented in Fig. 2, which provided predefined domains, measures, and criteria to guide interpretation [11–14].

## Results

### Recruitment

Three NATO member countries contributed Emergency Medical Teams to the study. A total of 25 healthcare professionals participated, including physicians, anaesthesiologists, nurses, and radiographers. Two of the participating teams were multinational. Most participants (92%) reported extensive prior experience in emergency trauma care, and 96% reported no prior experience using Extended Reality (XR) for training. All individuals invited to participate consented through their national command structures.

### Completion

All 25 participants completed the XR trauma simulation and the post-session survey, resulting in a 100% completion rate. No sessions were terminated prematurely, and no technical failures resulted in participant withdrawal.

### Acceptability

**Fig. 3:**
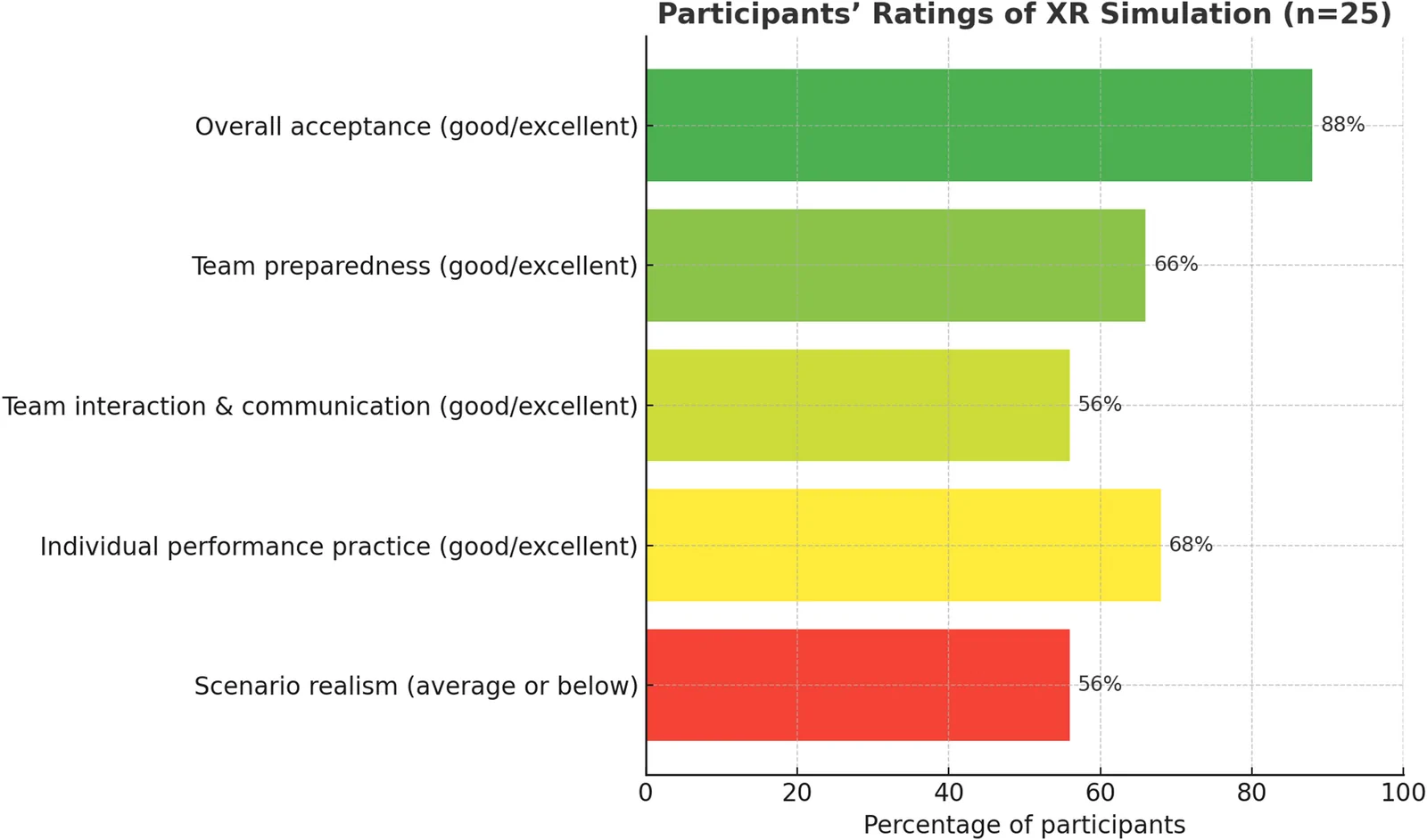
Participants’ ratings of XR trauma simulation (*n* = 25)

Overall, 88% of participants rated the XR trauma simulation as good or excellent for emergency trauma training (Fig. 3). The same proportion (88%) indicated willingness to use XR in future training activities. Qualitative responses reflected positive perceptions of XR as a learning modality, including perceived value for understanding team roles. One participant commented:

“…Great way to get an insight in different roles and responsibilities…” (Participant 2).

As shown in Fig. 3, overall acceptance of the XR trauma simulation was high, with 88% of participants rating the experience as good or excellent. Perceived educational value was strongest for clinical decision-making and preparedness, while ratings of scenario realism were more variable.

### Usability

Participants reported variable usability experiences. Challenges described in survey responses included headset navigation, manipulation of holographic elements, and interface intuitiveness. Lack of tactile feedback during invasive procedures, such as chest decompression and airway management, was also reported.

Survey ratings indicated that most participants evaluated usability as good or excellent, while a smaller proportion reported neutral or negative experiences.

### Perceived educational value

Participants reported perceived educational value of XR at both individual and team levels. Two-thirds (66%) rated XR as good or excellent for supporting teamwork and preparedness, while 56% rated it positively for supporting communication and decision-making within the team. At the individual level, 68% rated XR as good or excellent for providing a safe environment to practise and reflect on clinical decision-making.

Qualitative responses described XR as a potential adjunct to live exercises and noted opportunities for rehearsal of scenarios in resource-limited contexts.

### Barriers and areas for improvement

Participants identified several barriers related to the XR experience. More than half (56%) rated scenario realism as average or below, citing limited physiological fidelity and partial misalignment with Tactical Combat Casualty Care (TCCC) guidance. Some participants reported limited role differentiation within the XR environment, noting difficulty distinguishing profession-specific responsibilities. A recurring theme in qualitative responses was the absence of haptic or tactile feedback during procedural tasks, as well as challenges related to headset navigation and controls.

One participant noted:

“…Team mixed reality would improve teamwork…” (Participant 3)

## Discussion

This study addresses priorities articulated in the WHO Emergency Medical Teams (EMT) 2030 Strategy [1] and in contemporary simulation research [3–8] by examining the educational feasibility of Extended Reality (XR) trauma simulation within an authentic multinational training environment. By embedding XR within a large-scale civil–military live exercise, the study responds to the need for scalable, repeatable, and context-sensitive educational approaches that can complement resource-intensive live simulation and support workforce preparedness across diverse operational settings.

### Principal findings

This educational feasibility study demonstrated that XR trauma simulation could be implemented within a complex multinational exercise and was perceived by participants as acceptable and educationally valuable. Recruitment and completion were achievable within the operational constraints of the exercise, and participants expressed willingness to engage with XR in future training. Acceptability was high despite limited prior exposure to XR technology, suggesting openness to immersive modalities among experienced emergency care professionals.

Usability findings were more mixed. While participants were able to engage with the platform following a brief orientation, reported challenges related to interface navigation, lack of tactile feedback, and limitations in role differentiation constrained immersive fidelity. These findings are consistent with the developmental stage of current XR technologies and reflect challenges reported in prior studies of immersive simulation in emergency and trauma education [17, 18]. Importantly, participants described XR as supporting decision-making and team interaction in a psychologically safe environment, highlighting its potential educational value as a complement to, rather than a replacement for, live simulation.

From an educational perspective, XR aligns with experiential learning theory by enabling learners to engage in situated, high-stakes decision-making followed by structured reflection [19]. The immersive environment supports focused engagement while reducing physical risk, potentially mitigating cognitive load during complex scenarios [17]. XR may therefore function as a bridge between traditional simulation and clinical practice by supporting rehearsal, reflection, and team-based reasoning in contexts where access to live simulation is limited [17–24].

### Synthesis with the educational feasibility framework

Interpreted through established educational feasibility frameworks [11–13], the findings indicate feasibility across key domains, including recruitment, implementation, acceptability, usability, and perceived educational value. At the same time, identified barriers related to physiological fidelity, role differentiation, and technological maturity underscore the importance of iterative development prior to broader adoption. These findings reinforce the value of feasibility research as a necessary step in evaluating emerging educational technologies before progressing to formal efficacy or effectiveness studies.

Embedding XR within a live multinational exercise represents a distinctive contribution to the simulation literature. Feasibility studies are most informative when conducted under authentic conditions that expose contextual constraints and user interactions that may not be evident in controlled simulation-centre settings [14]. By examining XR deployment within an operationally complex environment, this study extends understanding of how immersive educational technologies function in practice and contributes evidence relevant to educators and programme leaders responsible for simulation design and integration.

### Comparison with existing literature

The findings are consistent with previous studies reporting that immersive and virtual trauma simulations can enhance perceived preparedness, teamwork, and decision confidence during live or hybrid training exercises [16, 17] . They also align with systematic reviews demonstrating the value of simulation-based education for developing both technical and non-technical skills essential to trauma and disaster response [18, 19] . However, much of the existing XR literature is derived from undergraduate cohorts or controlled pilot studies, leaving limited evidence regarding feasibility and educational integration among practising healthcare professionals in authentic operational contexts [3, 4, 8].

By extending feasibility evidence beyond simulation-centre environments, this study addresses a recognised gap in the literature concerning the deployment of XR within real-world, interprofessional, and multinational training settings. The ecological validity afforded by the live exercise context strengthens the relevance of these findings while also highlighting persistent challenges related to scalability, cost, and curricular alignment that must be addressed before XR can be integrated more widely into trauma education programmes [18, 19].

### Implications for educators

For simulation educators and programme leaders, these findings suggest that XR may function as a complementary modality within blended trauma education, offering opportunities for scalable rehearsal, distributed learning, and interprofessional engagement. XR may be particularly useful in contexts where access to live simulation is constrained by cost, logistics, or geography. However, educational value is contingent on careful pedagogical design, integration within curricula, and the use of structured debriefing to support reflection and learning.

Educators should critically appraise where XR adds value relative to traditional simulation, ensuring alignment with learning objectives, competency frameworks, and assessment strategies. Evidence-informed instructional design, drawing on experiential learning theory [20], cognitive load theory [25], and collaborative learning models, may support more effective integration of XR into trauma education and faculty development initiatives.

### Limitations

Several limitations should be considered when interpreting these findings. The sample size was appropriate for an educational feasibility study but limits generalisability and precludes inferential analysis. Recruitment through national command structures may have introduced selection bias, and findings were based primarily on self-reported perceptions rather than objective measures of performance or learning transfer. The post-session survey was purpose-designed for feasibility evaluation and was not psychometrically validated, which limits the strength of inferences that can be drawn.

The absence of longitudinal follow-up restricts insight into retention and transfer of learning to clinical practice. Additionally, the multinational military context introduces organisational, cultural, and linguistic factors that may influence engagement and usability and may limit direct transferability to civilian educational settings. Finally, the XR platform remains under development, with limitations in physiological fidelity, interactivity, and haptic feedback that influenced user experience.

## Conclusion

This study demonstrates that Extended Reality (XR) trauma simulation is educationally feasible and highly acceptable when embedded within a complex, multinational live simulation exercise. Recruitment, implementation, and completion targets were successfully achieved under operational conditions, and participants reported strong perceived educational value, particularly for clinical decision-making, teamwork, and reflective learning in a psychologically safe environment. While usability challenges related to realism, interface design, and tactile fidelity were identified, these limitations reflect the current developmental stage of XR technology rather than lack of educational potential.

Taken together, the findings support XR as a complementary modality to live simulation for interprofessional trauma education, particularly in contexts where scalability, access, and resource constraints limit traditional approaches. Future research should progress from feasibility to efficacy by examining the impact of XR on objective educational outcomes, including clinical decision-making, procedural performance, and teamwork behaviours, using validated assessment tools. Further work exploring curricular integration, cost-effectiveness, and scalability across civilian and military contexts is warranted, alongside continued technological development in areas such as haptics, adaptive physiology, and role-specific interaction design to enhance educational fidelity and support broader adoption.

## Supplementary Information

Supplementary Material 1.

Supplementary Material 2.

## Data Availability

The datasets generated and analysed during the current study are available from the corresponding author on reasonable request.
