# Supplementary material for: Feasibility and perceived educational value of extended reality trauma simulation for emergency medical teams in a multinational training context
Source: Adv Simul (Lond). 2026 Aug 18;11:86. doi: 10.1186/s41077-026-00418-x (PMC13760739; doi:10.1186/s41077-026-00418-x)
Supplement: Supplementary file 1 [file 41077_2026_418_MOESM1_ESM.pdf]

Structured Session Log Protocol

Educational Feasibility Framework – XR Trauma Simulation Study

Session Information

	Date	Location
	Team Identifier	Number of Participants
Represented	Session Duration (min)	Technical Setup Time (min)

Feasibility Domain 1: Recruitment and Completion

Indicator	Notes / Data
Participants scheduled vs. attended	
Dropouts / incomplete sessions	
Participant engagement observed	

Feasibility Domain 2: Implementation Fidelity

Indicator	Observation / Notes
Adherence to planned scenario steps	
Technical issues encountered	
Disruptions or environmental challenges	
Resolution method and time	

Feasibility Domain 3: Acceptability

Indicator	Observation / Notes
Participant engagement and immersion	
Observable frustration or discomfort	
Debrief comments indicating satisfaction or relevance	

Feasibility Domain 4: Usability

Indicator	Observation / Notes
Setup and calibration ease	
Navigation and interaction with XR interface	
Frequency of facilitator intervention	
Participant adaptability to XR controls	

Feasibility Domain 5: Educational Value

Indicator	Observation / Notes
Teamwork and role clarity	
Clinical reasoning or decision-making	
Verbalisation of learning points during debrief	
Realism of case perceived by participants	

Feasibility Domain 6: Barriers and Facilitators

Category	Observation / Notes
Technical	
Operational	
Educational	
Human Factors	
Facilitators	

Summary / Researcher Reflection

Key successes from this session	
Key challenges or barriers encountered	
Recommended adjustments for next session	
General impression of feasibility	
Observer(s)	Signature / Date
