# Supplementary material for: Feasibility and perceived educational value of extended reality trauma simulation for emergency medical teams in a multinational training context
Source: Adv Simul (Lond). 2026 Aug 18;11:86. doi: 10.1186/s41077-026-00418-x (PMC13760739; doi:10.1186/s41077-026-00418-x)
Supplement: Supplementary file 2 [file 41077_2026_418_MOESM2_ESM.pdf]

ACCEPTANCE OF VIRTUAL REALITY MODEL SURVEY

Thank you so much for your participation. This survey aims to gather insights into participants' backgrounds, professional experiences, and attitudes towards the integration of virtual simulation models in trauma management education.

NATO Live Simulation VW24 3rd to 7th of May 2024

For any enquiries and information please contact:
Dr Linda Sonesson linda.sonesson@kcl.ac.uk

BACKGROUND

1. **What is your nationality?** ____________________

2. **Are you educated in trauma management?** ____________________

3. **How many years have you been working?** ____________________

4. **Are you educated in trauma managemen?** ____________________

5. **Are you experienced in trauma management?** ____________________

ACCEPTANCE OF VIRTUAL SIMULATION MODEL

6. Have you been using virtual scenarios in trauma training before? Yes ○ No ○

PLEASE RATE THE QUESTIONS BELOW	SCALE OF 1 (VERY POOR) TO 5 (EXCELLENT)				
	1	2	3	4	5
**7. Please rate your experience of the virtual simulated case**	○	○	○	○	○
**8. How would you rate the virtual case as support for education and training in decision making of managing trauma patient?**	○	○	○	○	○
**9. How would you rate virtual simulation cases as part of preparation of trauma team training?**	○	○	○	○	○
**10. How would you rate virtual simulation supporting team interaction and communication?**	○	○	○	○	○
**11. How would you rate virtual simulation supporting team cohesion?**	○	○	○	○	○
**12. How would your rate virtual simulation supporting assessment of team performance in decision making of trauma injured patient?**	○	○	○	○	○
**13. How would you rate virtual simulation supporting assessment of individual performance in decision making of trauma injured patient?**	○	○	○	○	○
**14. How would you rate the realism of the virtual case?**	○	○	○	○	○
**15. How would you rate your user experience of the technology?**	○	○	○	○	○
**16. How likely is it that you would make use of virtual cases as part of training if you had the option to?**	○	○	○	○	○

17. Any other comments
